# CSF levels of Chitinase3like1 correlate with early response to cladribine in multiple sclerosis

**DOI:** 10.3389/fimmu.2024.1343892

**Published:** 2024-02-09

**Authors:** Damiano Marastoni, Matteo Foschi, Chiara Eccher, Francesco Crescenzo, Valentina Mazziotti, Agnese Tamanti, Albulena Bajrami, Valentina Camera, Stefano Ziccardi, Maddalena Guandalini, Francesca Bosello, Daniela Anni, Federica Virla, Ermanna Turano, Michele Romoli, Raffaella Mariotti, Francesca Benedetta Pizzini, Bruno Bonetti, Massimiliano Calabrese

**Affiliations:** ^1^ Neurology B, Department of Neurosciences, Biomedicine and Movement Sciences, University of Verona, Verona, Italy; ^2^ Neurology Unit, Department of Neuroscience, Multiple Sclerosis Center, S. Maria delle Croci Hospital, AUSL, Romagna, Ravenna, Italy; ^3^ Department of Biotechnological and Applied Clinical Sciences, University of L’Aquila, L’Aquila, Italy; ^4^ Neurology Unit, “Mater Salutis” Hospital, Scaligera AULSS 9, Verona, Italy; ^5^ Eye Clinic, Department of Surgery, Dentistry, Maternity, and Infant, University of Verona, Verona, Italy; ^6^ Department of Neurosciences, Biomedicine and Movement Sciences, University of Verona, Verona, Italy; ^7^ Neurology and Stroke Unit, Ospedale “Bufalini”, Cesena, Italy; ^8^ Radiology and Neuroradiology Unit, Azienda Ospedaliera Universitaria Integrata, Verona, Italy; ^9^ Neurology A, Azienda Ospedaliera Universitaria Integrata, Verona, Italy

**Keywords:** relapsing multiple sclerosis, cytokines, chemokines, cladribine, disease activity, biomarkers

## Abstract

**Background:**

Cladribine has been introduced as a high-efficacy drug for treating relapsing-remitting multiple sclerosis (RRMS). Initial cohort studies showed early disease activity in the first year after drug initiation. Biomarkers that can predict early disease activity are needed.

**Aim:**

To estimate cerebrospinal fluid (CSF) markers of clinical and radiological responses after initiation of cladribine.

**Methods:**

Forty-two RRMS patients (30F/12M) treated with cladribine were included in a longitudinal prospective study. All patients underwent a CSF examination at treatment initiation, clinical follow-up including Expanded Disability Status Scale (EDSS) assessment, and a 3T MRI scan after 6,12 and 24 months, including the evaluation of white matter (WM) and cortical lesions (CLs). CSF levels of 67 inflammatory markers were assessed with immune-assay multiplex techniques. The ‘no evidence of disease activity’ (NEDA-3) status was assessed after two years and defined by no relapses, no disability worsening measured by EDSS and no MRI activity, including CLs.

**Results:**

Three patients were lost at follow-up. At the end of follow-up, 19 (48%) patients remained free from disease activity. IFNgamma, Chitinase3like1, IL32, Osteopontin, IL12(p40), IL34, IL28A, sTNFR2, IL20 and CCL2 showed the best association with disease activity. When added in a multivariate regression model including age, sex, and baseline EDSS, Chitinase 3 like1 (p = 0.049) significantly increased in those patients with disease activity. Finally, ROC analysis with Chitinase3like1 added to a model with EDSS, sex, age previous relapses, WM lesion number, CLs, number of Gad enhancing lesions and spinal cord lesions provided an AUC of 0.76 (95%CI 0.60-0.91).

**Conclusions:**

CSF Chitinase 3 like1 might provide prognostic information for predicting disease activity in the first years after initiation of cladribine. The drug’s effect on chronic macrophage and microglia activation deserves further evaluation.

## 1 Introduction

Relapsing-remitting multiple sclerosis (RRMS) is characterized by the occurrence of new neurological symptoms with or without disability accumulation, followed in most cases by a slow collection of irreversible disability that defines the transition towards the progressive stage (secondary progressive MS, SPMS) (1, 2).

No definitive cure is available for MS, but current literature suggests that an early and proper introduction of a high-efficacy disease-modifying treatment (DMT) permits reducing disease activity in the first years after a diagnosis of MS and preventing long-term disability accumulation. An early introduction of a high-efficacy drug for treating MS has been suggested as the key to better controlling subsequent disease evolution (3–5). Notably, the introduction of many high-efficacy therapies raises concerns about their different effects and safety profile, in line with the need for a personalized approach to the treatment decision (6).

Along with clinical, demographical and MRI variables capable of predicting the treatment response, an approach based on individual biological and immunological characteristics has been suggested to provide additional value (7, 8).

Cladribine is an oral pulsed immune reconstitution therapy currently licensed for RRMS treatment, providing evidence of high efficacy in phase III trials (9–11) and real-life studies (12, 13). It is administered intermittently in two treatment courses over two years to produce long-term immunological effects requiring no further treatment for the next two years (14). Its mechanism of action involves the depletion of memory B cells, one of the immunological drivers of the disease (15). B cell recovery occurs slowly and in the presence of adequate T cell regulation without an increased risk of secondary autoimmunity (16). Initially, data from the CLARITY study indicated that 2.5% of patients treated with cladribine required a change in treatment due to disease activity after only one year of treatment (9). Subsequent studies have reported a frequency of clinical relapse and MRI activity, up to 22% (17) and 17% (18) in the first year of cladribine. Other studies evaluated possible prognostic factors for relapses in the first year of cladribine’s treatment.

*Post-hoc* analyses from the CLARITY study reported a better response in patients with “high disease activity” in the year before cladribine started, with ‘‘high disease activity’’ defined by the occurrence of two relapses or one relapse and MRI activity while on therapy with another DMT, in the year before cladribine started (11).

In other studies, among others, a higher baseline annualized relapse rate, higher gadolinium-enhancing lesions count at baseline, higher baseline EDSS score, and higher number of DMTs before switching to cladribine have been suggested as factors related to disease activity on cladribine treatment (13, 19, 20). These observations, underline the importance of proper early patient selection.

Beyond clinical and MRI variables, fluid biomarkers able to predict early disease activity after drug initiation could be helpful. We herein report the interim results of a longitudinal prospective trial, with the aim to evaluate inflammatory cerebrospinal fluid markers as candidate predictors of early disease activity after cladribine initiation.

## 2 Materials and methods

### 2.1 Study population and design

Forty-two patients with RRMS, defined according to revised McDonald Criteria (2), who started cladribine at the MS Centre of Verona University Hospital, were recruited to participate in the CLAD19 clinical trial, a phase IV longitudinal prospective study.

Along with a diagnosis of RRMS, inclusion criteria were the absence of any other inflammatory disease and the availability of at least 1 ml of CSF before treatment initiation.

All patients underwent neurological evaluation, including the Expanded Disability Status Scale (EDSS) assessment (21), every six months, with additional examinations in case of relapses, and completed a two-years follow-up. A relapse was defined as a worsening of neurological impairment or appearance of a new symptom or abnormality attributable to MS, lasting at least 24 hours and preceded by the stability of at least one month (22). All patients were scheduled to undergo a brain and spinal cord 3T-MRI after 6 (re-baseline), 12 and 24 months after cladribine initiation. Adverse events, including the occurrence of lymphopenia, were recorded. Lymphopenia was defined in accordance with the Common Terminology Criteria for Adverse Events (CTCAE version 5.0) as follows: grade 0 (≥910 × 109/L), grade 1 (≥800 × 109/L), grade 2 (<800–500 × 109/L), grade 3 (<500–200 × 109/L), and grade 4 (<200 × 109/L). The combined three-domain status of ‘No evidence of disease activity (NEDA-3) was defined by no evidence of relapses, MRI activity (new or enlarged white matter T2 hyperintense lesions, Gadolinium enhancing lesions, Gd+), and 6-months confirmed disability progression (CDP), defined as an increase of ≥1 point in EDSS (23). The absence of new cortical lesions (CLs) was included in the definition of NEDA-3.

### 2.2 Ethical approval

The local ethics committee of the University of Verona approved the study (CLAD19 Study, CESC n°2018-004947-21), and informed consent was obtained from all the patients.

### 2.3 CSF protein analysis

CSF samples were obtained at the time of diagnosis, at least two months after the last relapse, according to Consensus Guidelines for CSF and Blood Biobanking (24). After centrifugation, the supernatant was stored separately at −80°C. Two independent investigators optimized and performed the CSF analysis, blinded to the patient’s clinical and MRI features. The concentrations (ng/mL/mgProt) of 67 inflammatory markers were assessed using immune-assay multiplex techniques based on the Luminex technology (Bio-Plex-X200 System equipped with a magnetic workstation; BioRad, Hercules, CA; Bio-Plex Pro Human Inflammation Assay, 37-plex screening panel and Bio-Plex Pro Human Chemokine Panel Assays 40-plex panel) according to previously published procedures (7, 25). The presence of CSF oligoclonal bands (OCB) and CSF/serum albumin ratio were assessed in each patient.

### 2.4 MRI acquisition protocol

All Brain and spinal cord MRI scans were acquired using a Philips Achieva 3T Scanner at the Neuroradiology Unit of the University Hospital of Verona. A manual quality check was carried out to exclude significant artefacts.

A standardized protocol was employed to acquire the following sequences: 1. 3D-T1 weighted Turbo Field Echo (TFE) (Repetition Time (TR)/Echo Time (TE) = 8.4/3.7 ms, voxel size of 1x1x1 mm, acquisition time of 5:51 minutes); 2. 3D-Double Inversion Recovery (DIR, TR/TE = 5500/292 ms, Inversion Times (TI) TI1/TI2 = 525/2530 ms voxel size of 1x1x1 mm, acquisition time of 10:49 minutes); 3. 3D-Fluid Attenuated Inversion Recovery (FLAIR) (TR/TE = 5500/292 ms, TI = 1650 ms voxel size of 1x1x1 mm, acquisition time of 4:48 minutes); 4. 3D-T1 weighted TFE post-contrast with the same parameters of the pre-contrast sequence (TR/TE = 8.4/3.7 ms, voxel size of 1x1x1 mm, acquisition time of 5:51 minutes).

### 2.5 MRI analysis

*Lesion Detection.* The number of brain WM lesions (WMLn) at baseline and new and enlarging WM lesions at the end of the study were assessed on FLAIR images by a neuroradiologist with extensive experience in MS (FBP). The number of total cortical lesions (CLn) and the new CLs were assessed on DIR images based on recent recommendations (26). Owing to the suboptimal performance of the MRI in visualizing subpial lesions, the present analysis has taken into account mainly the intracortical and leukocortical lesions.

### 2.6 Statistical analysis

Differences among groups (patients with and without disease activity in the two-year follow-up) were initially assessed with the Mann-Whitney and Chi-Square/Fisher exact tests when appropriate.

Random Forest (RF) analysis, a feature selection technique, was applied to identify clinical and radiological variables as well as the baseline CSF molecules most associated with and best discriminate between stable participants and those that developed disease activity by the end of the follow-up. Lower Minimal Depth (MD) values reported by a variable indicate higher predictive accuracy, while higher times a root measure indicates a higher predictive power. We split the cohort into training (80%) and testing (20%) sets. We fit the RF (1000 trees, 8 variables tried at each split) on the training set and used the testing set to evaluate its performance.

Both univariate and multivariate logistic regression models to determine the association between the baseline, statistically significant CSF variables and NEDA-3 events at 2 years, including clinical and demographical variables, were used.

The receiver operating characteristic (ROC) analysis (Youden index method) was used to identify the selected molecules’ cut-off that maximises the specificity and sensitivity of identifying patients with disease activity. An area Under Curve (AUC) with 95% Confidence Interval (CI) was reported.

A p-value <0.05 was considered statistically significant. Statistical analysis was performed by means of the R studio 3.5.3 version.

### 2.7 Data availability

Deidentified data will be shared on reasonable request from a qualified investigator.

## 3 Results

### 3.1 Patient’s cohort

Three patients were lost at follow-up. Demographic and clinical characteristics of the study population at baseline are reported in **Table 1**.

**Table 1:** Baseline demographic, clinical and MRI characteristics of the whole population and accordingly to disease activity after the second year of treatment.

	Total MS (n = 39)	EDA (n = 20)	NEDA (n =19)	*p* value between EDA and NEDA
Age - yr	34 ± 11.0	32.20 ± 11.43	35.16 ± 11.14	0.298
Female– no. (%)	28 (71.8)	15 (75)	13 (68.42)	0.731
EDSS score - median (range)	2.0 (0.0-5.0)	1.5 (0.0-4.0)	2.0 (0.0-5.0)	0.123
Disease duration - mean ± SD	2.44 ± 4.3	3.25 ± 5.45	1.58 ± 2.36	0.439
Previous treatment – yes (%)	17 (43.6)	11 (55)	6 (31.58)	0.200
Relapses in previous year - mean ± SD	1.46 ± 0.55	1.4 ± 0.60	1.53 ± 0.51	0.430
Time from last relapse- months	2.82 ± 1.48	2.85 ± 1.50	2.79 ± 1.51	0.830
BMI (Kg/m^2^)	23.7 ± 2.1	23.9 ± 2.2	23.4 ± 2.1	0.143
WMLN - mean ± SD	11.15 ± 5.59	11.85 ± 5.52	10.42 ± 5.72	0.422
Spinal Cord lesion number - mean ± SD	1.23 ± 1.49	0.85 ± 1.09	1.63 ± 2.11	0.359
Gd+ lesions - mean ± SD	0.49 ± 0.91	0.55 ± 0.89	0.42 ± 0.96	0.583
CLN - mean ± SD	2.90 ± 4.96	3.95 ± 6.4	1.79 ± 2.46	0.280
CSF OCBs (yes/not)	32/7	17/3	15/4	0.695
Albumin CSF/serum	5.23 ± 2.1	5.01 ± 1.82	5.38 ± 2.41	0.939

EDA, evidence of disease activity; NEDA, no evidence of disease activity; EDSS, Expanded Disability Status Scale; WMLN, White Matter Lesion Number; Gd+ lesions, Gadolinium enhancing lesions; CLN, Cortical lesion number; CTh, Cortical Thickness; OCBs, Oligoclonal bands; CSF, cerebrospinal fluid.

Patients with disease activity had an increased EDSS at the time of diagnosis. A p value < 0.05 was considered significant.

Twenty-two patients (56%) began cladribine as the first disease modifying drug due to high disease activity with at least two relapses with disability accumulation and radiological activity in the year before. Seventeen patients (44%) were switched to cladribine from a previous first-line treatment (12 from dimethyl fumarate, 5 from interferon beta1a) due to the inefficacy.

**Figure 1:**
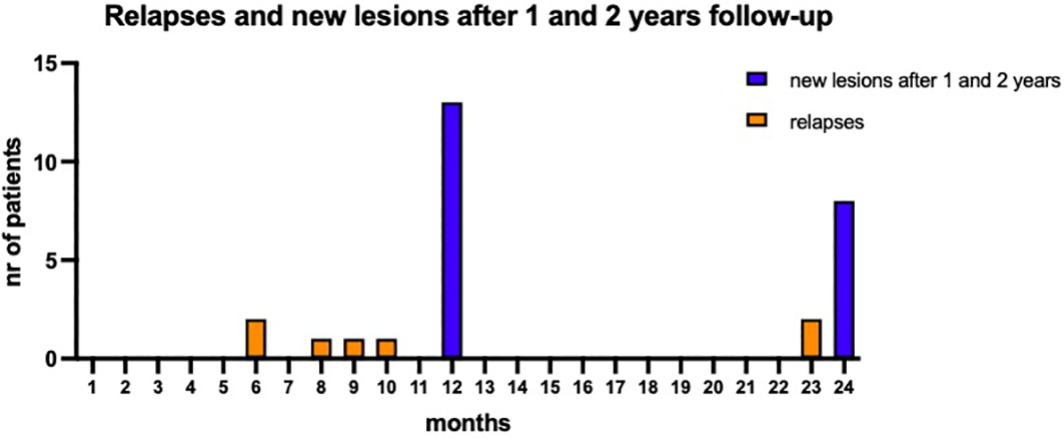
Disease activity during the two years of follow-up. Five patients experienced at least a relapse in the first year while the occurrence of new or enlarging T2 lesions, new CLs, or Gd enhancing lesions was evident in 13 patients after first year of follow-up. The clinical and radiological activity reduced over the second year of follow-up.

Disease activity was most evident during the first year of follow-up (**Figure 1**). Six patients (15%) experienced at least 1 relapse (five in the first year of treatment and only one in the second year). 46.2% of patients (18/39) showed new or enlarging T2 lesions, new CLs, or Gd-enhancing lesions: MRI activity was more common in the first year (13/39, 33%) compared to the second year (8/39, 21%); CDP occurred in 4 patients, with only one patient experiencing disability progression independent from relapses.

At the end of follow-up, 53.8% of patients (21/39) remained free from MRI activity while 48% of patients (19/39) remained free from disease activity (NEDA, **Table 1**).

No severe adverse drug reactions leading to discontinuation were reported; no grade 4 lymphopenia was reported to have occurred, and all patients had values above 800/mm3 after the first year of treatment. Two VZV reactivation events leading to antiviral therapy administration were reported in the first year of follow-up.

### 3.2 Markers associated with disease activity during the first year

After comparing levels of all molecules in both groups of patients (with and without disease activity within the previous year of treatment), sTNFR1, sTNFR2, sILR6a and sCD163 were significantly increased in the active group (**Supplementary Table 1**).

### 3.3 Markers associated with disease activity after two years

#### 3.3.1 Feature selection

Few molecules were increased in those patients experiencing disease activity after two years (**Table 2**): CCL24, CXCL2, CCL2, CXCL16 and Chitinase3like1.

**Table 2:** CSF cytokines and chemokines levels before cladribine administration in the whole population and accordingly to EDA and NEDA after two-year follow-up.

	Total MS (n = 39)	EDA (n = 20)	NEDA (n =19)	*p* value
CCL21	5964.03 ± 12230.36	4277.42 ± 5244.73	7739.41 ± 16740.98	0.9668
CXCL13	6.75 ± 10.06	5.71 ± 5.95	7.78 ± 13.06	0.7291
CXCL5	995.21 ± 751.75	1227.20 ± 933.86	751.01 ± 387.01	0.2957
CCL11	73.97 ± 84.13	95.88 ± 107.62	53.20 ± 47.78	0.3127
CCL24	33.92 ± 36.24	43.02 ± 46.64	24.35 ± 16.94	0.0893
CCL26	73.09 ± 93.97	83.90 ± 115.61	61.71 ± 65.33	0.8786
CX3CL1	350.67 ± 313.57	445.05 ± 396.98	251.33 ± 144.61	0.0742
GMCSF	65.77 ± 73.45	85.40 ± 88.98	45.11 ± 46.41	0.0696
CXCL1	151.11 ± 160.89	177.06 ± 197.82	123.80 ± 108.64	0.5686
CXCL2	50.12 ± 52.57	66.63 ± 68.49	35.56 ± 27.72	0.0357
CCL1	63.16 ± 65.63	73.12 ± 81.43	53.75 ± 46.47	0.5990
CXCL10	440.57 ± 587.73	571.44 ± 766.99	302.82 ± 263.07	0.3367
CXCL11	132.87 ± 783.75	254.08 ± 1093.92	5.28 ± 11.95	0.2923
CCL2	507.59 ± 787.23	711.63 ± 1059.49	292.81 ± 168.77	0.0305
CCL8	203.24 ± 829.83	329.07 ± 1139.46	70.79 ± 217.08	0.5133
CCL7	81.53 ± 69.64	90.69 ± 78.03	71.83 ± 60.35	0.7078
CCL13	32.71 ± 56.69	41.93 ± 67.31	23.50 ± 43.54	0.2014
CCL22	23.47 ± 26.41	27.92 ± 34.30	19.01 ± 14.68	0.5014
MIF	6583.69 ± 16129.92	4615.29 ± 9511.65	8655.70 ± 21093.18	0.5133
CXCL9	31.83 ± 43.02	46.15 ± 55.69	16.76 ± 12.87	0.1494
CCL3	6.75 ± 5.13	7.88 ± 6.06	5.55 ± 3.72	0.2138
CCL15	409.87 ± 297.29	483.80 ± 382.75	332.06 ± 139.57	0.4440
CCL20	2.11 ± 2.33	2.65 ± 3.01	1.62 ± 1.44	0.5551
CCL19	263.04 ± 268.81	398.37 ± 316.74	225.95± 209.32	0.6870
CCL23	9.83 ± 10.79	12.12 ± 13.67	7.21 ± 5.48	0.2196
CXCL16	2040.61 ± 1137.26	2410.59 ± 1308.50	1651.16 ± 781.63	0.0840
CXCL12	2006.14 ± 2843.47	2475.87 ± 3803.71	1511.68 ± 1137.45	0.4115
CCL25	574.15 ± 889.93	598.99 ± 1028.70	548.00 ± 743.98	0.7705
TNF	75.15 ± 70.87	87.96 ± 87.74	61.66 ± 45.86	0.5687
sTNFR1	5250.49 ± 3930.86	6042.08 ± 4598.56	4417.24 ± 2978.16	0.1576
sTNFR2	700.93 ± 530.89	859.85 ± 625.14	533.64 ± 353.41	0.1197
TWEAK	5356.56 ± 8842.29	5163.98 ± 7781.24	5559.28 ± 10053.04	0.8350
APRIL	115117.59 ± 90655.80	123213.02 ± 102989.76	106596.09± 77488.60	0.8134
BAFF	17953.14 ± 15213.18	21026.34 ± 18553.55	14718.19 ± 10183.25	0.2244
LIGHT	93.91 ± 239.99	135.61 ± 323.64	49.90 ± 84.04	0.8219
sCD30	2643.43 ± 2433.90	2833.03 ± 2472.73	2443.86 ± 2443.33	0.6267
IFN gamma	23.60 ± 24.83	27.05 ± 31.36	19.96 ± 15.38	0.9990
INF alfa2	40.49 ± 82.22	40.66 ± 75.23	40.35 ± 90.16	0.9864
IFN beta	26.60 ± 25.22	32.52 ± 31.33	19.32 ± 12.38	0.5097
IL28A	79.44 ± 274.64	130.59 ± 395.28	33.67 ± 55.58	0.6163
IL29	65.55 ± 76.27	76.49 ± 95.89	54.62 ± 50.74	0.7804
sIL6R beta	118081.52 ± 81401.00	128098.59 ± 85099.86	107537.23 ± 78200.47	0.5315
IL1 beta	2.95 ± 3.86	3.56 ± 4.85	2.30 ± 2.39	0.7024
IL4	27.78 ± 26.02	33.95 ± 33.12	21.61 ± 14.65	0.3854
IL6	29.90 ± 52.40	28.33 ± 41.25	31.69 ± 64.25	0.1532
IL8	67.20 ± 95.04	91.09 ± 125.75	42.05 ± 32.78	0.6071
IL10	16.41 ± 15.90	20.49 ± 19.36	12.11 ± 10.02	0.2325
IL16	76.23 ± 125.31	96.41 ± 162.86	55.00 ± 65.01	0.2829
sIL6Ra	5499.32 ± 3375.99	6026.98 ± 3543.83	4943.89 ± 3188.98	0.1749
IL11	3.11 ± 3.41	3.22 ± 3.94	3.03 ± 3.05	0.8463
IL12(p40)	33.55 ± 40.19	40.12 ± 53.12	28.22 ± 26.28	0.9999
IL12(p70)	15.07 ± 26.74	19.72 ± 31.19	10.94 ± 22.16	0.4687
IL19	231.79 ± 266.31	243.32 ± 315.95	219.54 ± 210.98	0.9010
IL20	39.56 ± 64.89	47.14 ± 84.82	31.99 ± 36.60	0.4699
IL22	61.84 ± 79.01	80.06 ± 106.19	44.58 ± 34.49	0.2844
IL26	2250.08 ± 2625.67	2477.70 ± 3250.15	1995.68 ± 1751.23	0.9004
IL27	187.22 ± 234.59	242.29 ± 320.76	136.38 ± 99.62	0.9787
IL32	104.68 ± 127.53	141.32 ± 164.74	63.96 ± 41.66	0.1579
IL34	796.09 ± 768.15	865.57 ± 996.56	726.60 ± 459.84	0.6279
IL35	209.09 ± 186.10	251.38 ± 221.92	164.31 ± 130.89	0.4626
MMP1	548.47 ± 1026.25	672.98 ± 1283.33	390.00 ± 580.79	0.9786
MMP2	1118.42 ± 1362.71	1218.66 ± 1655.24	984.78 ± 861.71	0.7666
Osteocalcin	883.54 ± 620.69	921.08 ± 621.22	844.02 ± 634.62	0.7029
Osteopontin	113372.41 ± 82555.24	117991.91 ± 87616.25	108509.79 ± 78969.88	0.9226
Pentraxin3	351.42 ± 267.70	360.22 ± 334.07	342.15 ± 182.50	0.4115
TSLP	29.27 ± 28.57	35.07 ± 36.14	22.79 ± 15.20	0.2851
sCS163	59394.53 ± 34319.58	66735.91 ± 37941.27	51666.76 ± 29053.45	0.1937
Chitinase3like1	58668.44 ± 45223.32	72609.30 ± 51483.53	43993.75 ± 32809.67	0.0573

Values are expressed as ng/ml/mgProt; mean ± SD are reported. A p value < 0.05 at Mann-Whitney test was considered significant.

CSF, cerebrospinal fluid; EDA, evidence of disease activity; NEDA, no evidence of disease activity; CCL21, chemokine C-C motif ligand 21; CXCL13, chemokine C-X-C motif ligand 13; CXCL5, chemokine C-X-C motif ligand 5; CCL11, chemokine C-C motif ligand 11; CCL24, chemokine C-C motif ligand 24, CCL26, chemokine C-C motif ligand 26; CX3CL1, chemokine C-X3 -C motif ligand 1; GMCSF, Granulocyte macrophage colony-stimulating factor; CXCL1, chemokine C-X-C motif ligand 1; CXCL2, chemokine C-X-C motif ligand 2; CCL1, chemokine C-C motif ligand 1; CXCL10, chemokine C-X-C motif ligand 10; CXCL11, chemokine C-X-C motif ligand 11; CCL2, chemokine C-C motif ligand 2; CCL8, chemokine CC motif ligand 8; CCL7, chemokine C-C motif ligand 7; CCL13, chemokine C-C motif ligand 13; CCL22, chemokine C-C motif ligand 22; MIF, macrophage migration inhibitory; CXCL9, chemokine C-X-C motif ligand 9; CCL3, chemokine C-C motif ligand 3; CCL15, chemokine CC motif ligand 15; CCL19, chemokine C-C motif ligand 19; CCL23, chemokine C-C motif ligand 23; CXCL16, chemokine C-X-C motif ligand 16; CXCL12, chemokine C-X-C motif ligand 12 or stromal cell-derived factor; CCL25, chemokine C-C motif ligand 25; TNF, tumor necrosis factor; sTNFR1, soluble receptor 1 of tumor necrosis factor; sTNFR2, soluble receptor 2 of tumor necrosis factor; TWEAK, TNF-like weak inducer of apoptosis; APRIL, A proliferation-inducing ligand, or tumor necrosis factor ligand superfamily member 13; BAFF, B cell-activating factor of the tumor necrosis factor family; LIGHT, tumor necrosis factor ligand superfamily member 14 or tumor necrosis factor; sCD30, soluble form of CD30; IFNg, interferon gamma; IFNalfa2, interferon alfa 2; IL28a, interleujin-28a; sIL6R-beta, soluble receptor beta of interleukin-6; IL1beta, interleukin-1 beta; IL4, interleukin-4; IL6, interleukin-6; IL8, interleukin-8; IL10, interleukin-10; IL16, interleukin-16; sILRa, soluble interleukine receptor a; IL12 (p70), interleukin-12 (p70); IL20, interleukin-20; IL22, interleukin-22; IL 26, interleukin-26; IL32, interleukin-32; IL34, interleukin-34; IL35, interleukin-35; sCD163, Soluble form of CD163.

**Figure 2:**
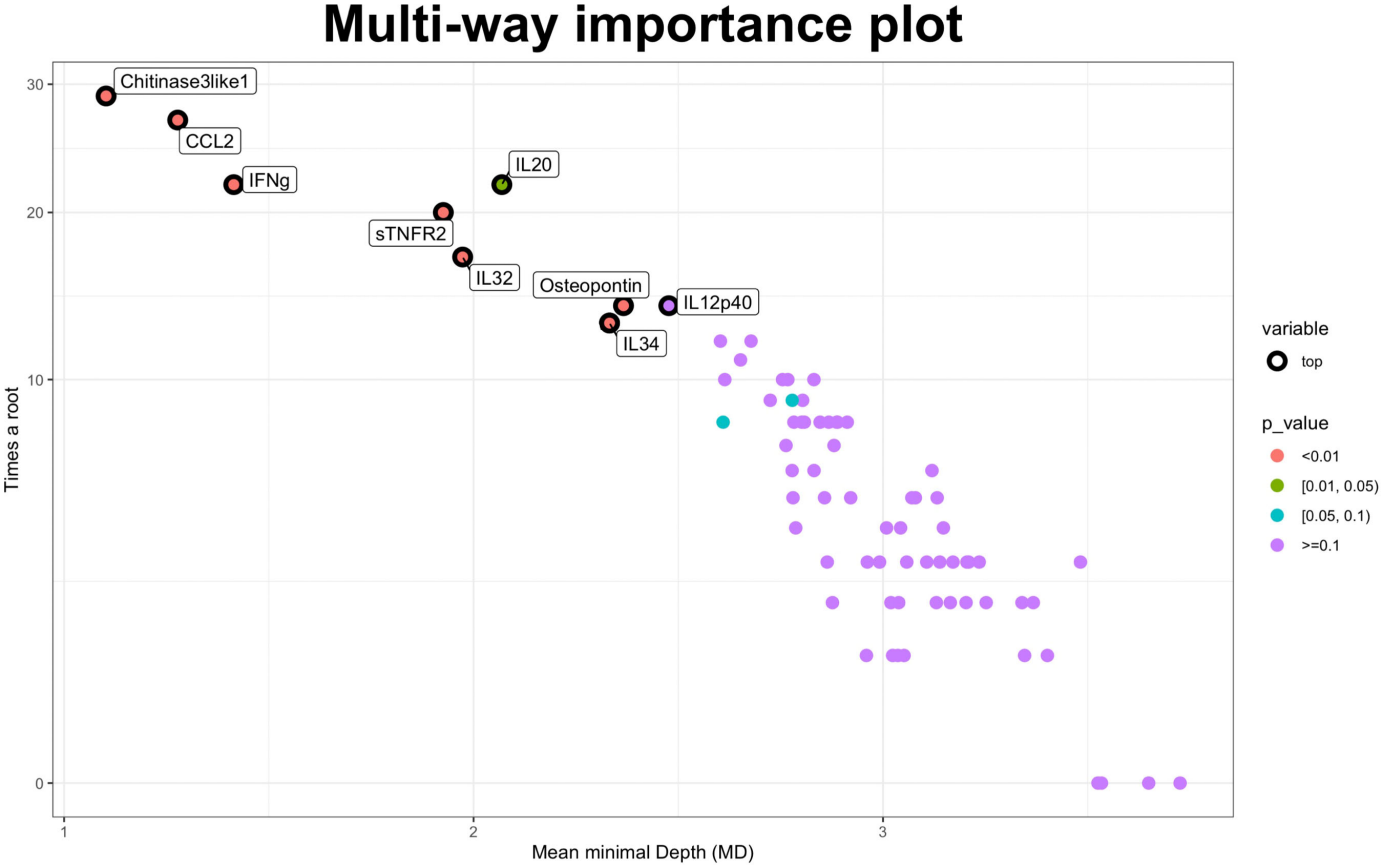
Random forest approach. Multiway importance plot: most important variables associated with the NEDA-3 status after two-years’ follow-up. Minimal Depth and times a root measures are showed. Lower Minimal Depth values indicate higher predictive accuracy, while higher times a root measure indicates a higher predictive power. NEDA-3, no evidence of disease activity-3.

The RF was then applied to identify the baseline CSF molecules to discriminate between EDA patients and stable participants based on a 2-year MRI measure. WMLn, CLn, number of Gadolinium enhancing lesions and spinal cord lesions, pre-treatment relapse frequency and presence of Oligoclonal bands were included in the analysis. Lower Minimal Depth (MD) values in variable reports indicate greater predictive accuracy, whereas increased values of times a root indicate higher predictive power. The cohort was split into training (80%) and testing (20%) sets. No significant differences have been found regarding disease activity, baseline clinical, demographical and radiological variables when comparing the training and the testing set (not shown). The RF was fitted on the training set, and the testing set was used to evaluate its performance. The model had an overall out-of-bag (OOB) error on the training set of 33% and achieved a prediction accuracy of 0.87 on the testing set. Based on the RF analysis, the most important predictors of EDA-3 in our cohort were Chitinase3like1, CCL2, and INFgamma, followed by sTNFR2, IL32, Osteopontin and IL34 (p<0.010). IL20 showed lower importance (p between 0.01 and 0.05) (**Figure 2**).

#### 3.3.2 Logistic regression analysis

Univariate logistic regression analysis on the significant molecules selected at RF suggested Chitinase3like1 as the best associated with 2-year EDA [β 1.78x10^-5^ (se 9.88x10^-6^), p = 0.071], along with sTNFR2 [β 142.19x10^-3^ (se 79.32x10^-5^), p = 0.073], and IL32 [β 0.01 (se 6.02x10^-3^), p = 0.080]. When added to a multivariate regression model that included age, sex, and baseline EDSS (AIC 57.113), Chitinase3like1 resulted significantly associated with disease activity [β 2.27x10^-5^ (se 1.15x10^-5^e-05), p = 0.049] after 2 years follow-up (AIC 60.723). Finally, logistic regression including Chitinase3like1 (as per percentile) and predefined factors such as Body Mass Index (BMI), prior DMT and time from last relapse confirmed the role of Chitinase3like1 as independent predictor of EDA (OR for 4^th^ percentile=0.44, 95%CI=0.003-0.72, p = 0.028).

#### 3.3.3 ROC analysis

**Figure 3:**
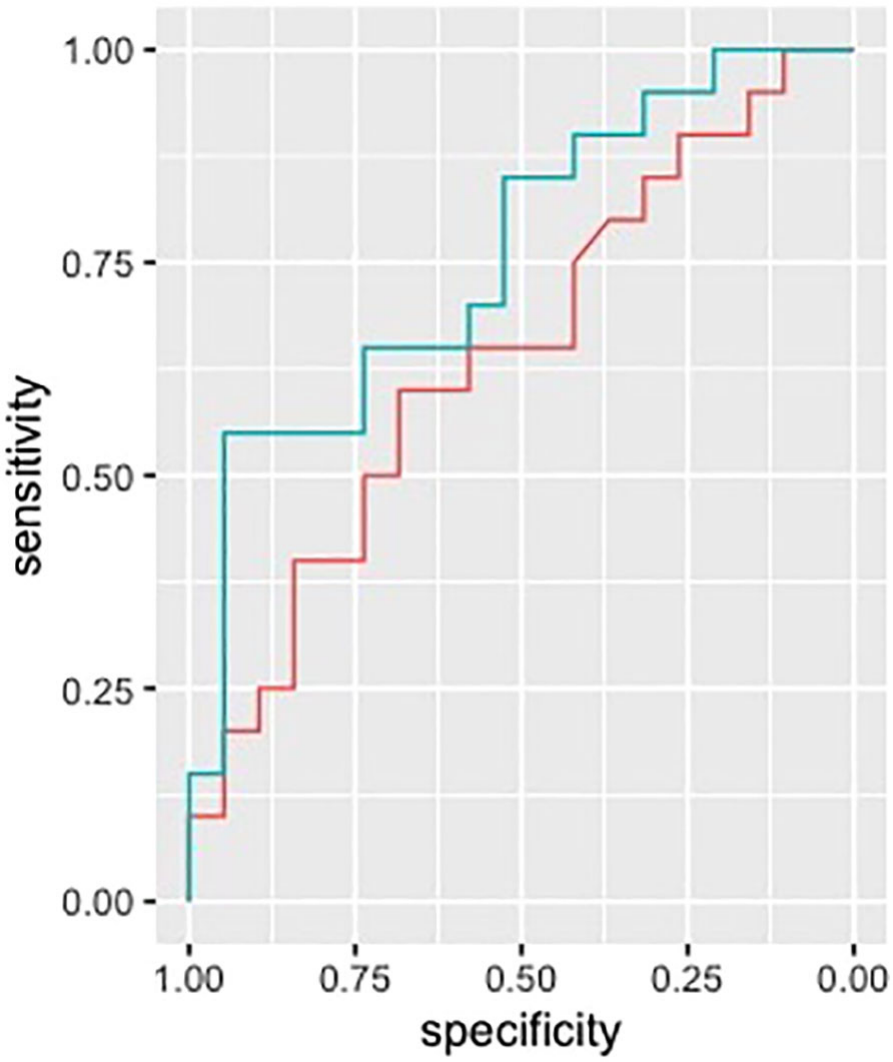
ROC analysis. ROC curves in discriminating between patients with and without disease activity are shown. CHIT3L1 (line green) improved the AUC (0.76, 95%CI 0.60-0.91) when added to a model with relapses in the previous year, WMLN, CLN, Gd+ lesions and spinal cord lesions. (line red, AUC 0.64, IC95% 0.46-0.82). ROC, receiver operating characteristic; CHIT3L1, Chitinase3like1; AUC, Area under the curve; WMLN, White matter lesion number; CLN, Cortical lesion number; Gd+ lesions, Gadolinium enhancing lesions.

ROC analysis performed on clinical and radiological variables provided an AUC of 0.64 (IC95% 0.46-0.82) when considering previous relapses, WMLn, CLn and number of Gad enhancing lesions and spinal cord lesions. Including EDSS, sex and age provided an AUC of 0.68 (IC95% 0.50-0.85). Finally, adding Chitinase3like1 to all the above-mentioned variables improved the performance of the analysis (AUC 0.76, 95%CI 0.60-0.91) (**Figure 3**).

## 4 Discussion

Establishing reliable fluid markers that could be helpful in the treatment selection process remains an unmet need. Interestingly, despite a high-efficiency treatment like cladribine, we provided evidence of increased levels of a few inflammatory markers, particularly Chitinase3like1, in patients with disease activity.

High-efficacy treatments are becoming of considerable interest in managing early disease phases since a proper early introduction of a high-efficacy treatment limits subsequent MS-related disability (27). Still, safety and efficacy concerns remain in line with the increasing number of available drugs with different mechanisms of action so far available (28).

In line with the pivotal study (9) our data confirm cladribine’s efficacy and safety profile. In the CLARITY trial, 80% of cladribine-treated patients remained relapse-free after two years (85% in our cohort). Unlike previous real-life studies, which reported a frequency of 17% (18) and 12% (20) in the years 1 and 2, respectively, here we report a higher rate of MRI activity (33% after year 1 and 46% after two years of follow-up). Overall, the proportion of patients who showed NEDA status after two years is slightly lower than reported in a large retrospective observational study (20) (48% vs 64%). Nevertheless, we confirm the higher efficacy of cladribine over the two years of follow-up when compared to disease activity, which is, in many cases, evident in the first year after drug introduction. Having included the appearance of new cortical lesions in the NEDA parameters and the use of a 3.0T MRI Scan may explain these discrepancies. Another significant point is the high-activity population selected, which included patients who switched from a first-line therapy due to inefficacy and patients who started cladribine as a first-line therapy due to high disease activity in the previous year according to regulatory agencies guidelines.

Our data show an absence of grade IV lymphopenia in accordance both with what was reported in clinical trials (< 1%) (9), and in an observational Italian study from 56 MS centers that reported that the risk of Grade 4 lymphopenia with cladribine is very lower (29).

CSF inflammatory markers could be helpful as they reflect chronic intrathecal processes that often are not adequately targeted by DMTs. In the case of cladribine, the drug’s capability to cross the blood-brain barrier raises the possibility that intrathecal inflammatory niches could be targeted by the drug and the peripheral immune compartment (30).

Notably, we did not find frequently significantly increased levels of many cytokines/chemokines among the 67 tested in those patients with disease activity. This could be due to the relatively low number of patients included and the administration of previous therapies in some patients (all the switches to cladribine were due to inefficacy of a first-line drug, i.e. dimethyl fumarate or interferon). Nevertheless, the relatively low rate of intrathecal inflammation in those patients with disease activity could be attributed to an intrathecal drug efficacy on the cellular innate and adaptive compartment.

In a previous study (8) we focused on CSF markers capable to predict response to a first-line disease modifying therapy like dimethyl fumarate, while we herein highlighted markers of response to a high-efficacy disease modifying therapy like Cladribine. When wishing to predict response to first-line therapies, patients with high disease activity show increased levels of many inflammatory molecules, often related to the TNF family (8, 31) or the B cell chemoattractant chemokines CXCL12 and CXCL13 (7, 32).

On the contrary, CSF Chitinase3like1 is a marker of microglia/macrophage activation linked to MS disease activity (33, 34) and disability accumulation (35).

CHI3L1 or YKL-40 is implicated in diverse pathologic conditions (34). Both an association with M1 and M2 macrophage differentiation with concurrent regulation of inflammation have been found (36), suggesting the molecule as involved in the balance between Th1/Th2 inflammatory response (37). Furthermore, its role involves tissue remodeling; accordingly, in the CNS, a role of CHI3L1 in modulating astrocytic and microglial reactive gliosis has been suggested (34). Notably, its expression has been recently associated with chronic inflammatory activity with macrophage/microglia activation with little astrocyte reactivity, that occurs on the edge of chronic active lesions, a candidate marker of disease severity (38). In such a context, CHIT3L1 has been considered a surrogate marker of intrathecal processes that drive disease progression and disability accumulation over the MS disease course (39, 40). Whether the intrathecal effectiveness of cladribine could affect these intrathecal inflammatory niches with particular regard to chronic microglia activation remains debated. This opens up the possibility that a partial drug efficacy occurs in the intrathecal compartment, or that chronic intrathecal inflammatory and neurodegenerative processes occur independently on high efficacy drug administration. Further studies will clarify this aspect, in line with the need to provide a proper high-efficacy therapy capable of acting on both peripheral and central nervous system compartments.

Accordingly, a combined molecular approach could provide informative ‘omics’ markers that could inform on drug mechanisms as well as help in providing a personalized treatment approach (41).

Our work is not without limitations. In particular, the low sample size, the absence of a validation cohort, the need for experimental replication, limit the conclusions that can be drawn. A further limitation stands on the a-priori definition for the application of Random Forest approach. This emerged *a priori* as the preferred way to handle the amount of data, covering both regression and classification tasks. Further studies will be needed to define the potential role and replicability of our findings with approaches handling larger and higher-dimensional data, such as Elastic net and support vector machine.

In the end, we tried to detect CSF inflammatory markers of disease activity after a high-efficacy treatment for MS.

This evidence: i) adds further value to Chitinase3like1 as a biomarker that could help clinicians through a personalized treatment approach; ii) suggests insights into cladribine mechanism of action: so far, a good efficacy on the peripheral compartment, and a possible efficacy on the intrathecal one have been suggested, however its effect on chronic macrophage and microglia activation deserves further evaluations.

## Data availability statement

The original contributions presented in the study are included in the article/**Supplementary Material**. Further inquiries can be directed to the corresponding author.

## Ethics statement

The Ethic Committee of the University of Verona approved the present study (Protocol number 66418). All participants provided written informed consent to the study.

## Author contributions

DM: Conceptualization, Data curation, Formal analysis, Investigation, Writing – original draft, Writing – review & editing. MF: Data curation, Formal analysis, Writing – original draft, Writing – review & editing. CE: Data curation, Writing – original draft, Writing – review & editing. FC: Data curation, Writing – review & editing. VM: Data curation, Writing – review & editing. AT: Data curation, Methodology, Software, Writing – review & editing. AB: Data curation, Writing – review & editing. VC: Data curation, Writing – review & editing. SZ: Data curation, Methodology, Writing – review & editing. MG: Data curation, Writing – review & editing. FB: Data curation, Writing – review & editing. DA: Data curation, Writing – review & editing. FV: Data curation, Writing – review & editing. ET: Data curation, Writing – review & editing. MR: Supervision, Writing – review & editing. RM: Data curation, Writing – review & editing. FP: Investigation, Methodology, Writing – review & editing. BB: Supervision, Writing – review & editing. MC: Conceptualization, Funding acquisition, Project administration, Resources, Supervision, Writing – review & editing.
