# Supplementary material for: CSF levels of Chitinase3like1 correlate with early response to cladribine in multiple sclerosis
Source: Front Immunol. 2024 Feb 9;15:1343892. doi: 10.3389/fimmu.2024.1343892 (PMC10885800; doi:10.3389/fimmu.2024.1343892)
Supplement: Supplementary file 1 [file Table_1.docx]

Supplementary Material

# Supplementary Tables

**Supplementary Table 1.** CSF cytokines and chemokines levels before cladribine administration in the whole population and accordingly to disease activity after one-year follow-up.

|  | **Total MS (n = 39)** | **NEDA (n=25)** | **EDA (n=14)** | **p value** |
| --- | --- | --- | --- | --- |
| CCL21 | 5964.03 ± 12230.36 | 6372.1 ± 14713.5 | 5235.3 ± 6056.2 | 0.346 |
| CXCL13 | 6.75 ± 10.06 | 6.8 ± 11.6 | 6.7 ± 6.7 | 0.429 |
| CXCL5 | 995.21 ± 751.75 | 879.8 ± 543.2 | 1201.4 ± 1017.8 | 0.784 |
| CCL11 | 73.97 ± 84.13 | 61.8 ± 58.9 | 96.5 ± 117.2 | 0.695 |
| CCL24 | 33.92 ± 36.24 | 27.6 ± 20.7 | 45.3 ± 53.2 | 0.289 |
| CCL26 | 73.09 ± 93.97 | 58.7 ± 67.0 | 98.8 ± 128.1 | 0.478 |
| CX3CL1 | 350.67 ± 313.57 | 284.6 ± 182.9 | 468.6 ± 449.8 | 0.239 |
| GMCSF | 65.77 ± 73.45 | 53.4 ± 59.2 | 87.8 ± 92.2 | 0.195 |
| CXCL1 | 151.11 ± 160.89 | 128.5 ± 137.4 | 191.5 ± 195.1 | 0.216 |
| CXCL2 | 50.12 ± 52.57 | 41.8 ± 36.5 | 68.3 ± 76.7 | 0.100 |
| CCL1 | 63.16 ± 65.63 | 51.3 ± 46.6 | 85.0 ± 89.3 | 0.236 |
| CXCL10 | 440.57 ± 587.73 | 408.9 ± 597.3 | 497.1 ± 587.9 | 0.426 |
| CXCL11 | 132.87 ± 783.75 | 200.5 ± 979.1 | 12.1 ± 34.6 | 0.919 |
| CCL2 | 507.59 ± 787.23 | 514.1 ± 942.3 | 496.0 ± 414.7 | 0.515 |
| CCL8 | 203.24 ± 829.83 | 253.6 ± 998.7 | 113.3 ± 397.2 | 0.761 |
| CCL7 | 81.53 ± 69.64 | 76.8 ± 60.3 | 89.6 ± 85.2 | 0.933 |
| CCL13 | 32.71 ± 56.69 | 25.9 ± 43.9 | 45.8 ± 76.0 | 0.564 |
| CCL22 | 23.47 ± 26.41 | 20.4 ± 16.6 | 29.3 ± 39.3 | 0.747 |
| MIF | 6583.69 ± 16129.92 | 6956.6 ± 18531.6 | 5917.8 ± 11214.2 | 0.081 |
| CXCL9 | 31.83 ± 43.02 | 21.7 ± 23.5 | 50.0 ± 61.9 | 0.303 |
| CCL3 | 6.75 ± 5.13 | 5.8 ± 3.6 | 8.5 ± 6.9 | 0.317 |
| CCL15 | 409.87 ± 297.29 | 350.3 ± 179.7 | 516.2 ± 424.0 | 0.317 |
| CCL20 | 2.11 ± 2.33 | 2.1 ± 2.1 | 2.0 ± 3.1 | 0.575 |
| CCL19 | 263.04 ± 268.81 | 239.1 ± 215.2 | 305.8 ± 350.2 | 1.000 |
| CCL23 | 9.83 ± 10.79 | 9.0 ± 7.5 | 11.5 ± 15.9 | 1.000 |
| CXCL16 | 2040.61 ± 1137.26 | 1804.5 ± 846.7 | 2462.3 ± 1469.1 | 0.317 |
| CXCL12 | 2006.14 ± 2843.47 | 1672.1 ± 1297.4 | 2602.6 ± 4464.2 | 0.784 |
| CCL25 | 574.15 ± 889.93 | 463.6 ± 663.1 | 771.6 ± 1199.1 | 0.718 |
| TNF | 75.15 ± 70.87 | 70.4 ± 59.3 | 83.6 ± 89.9 | 0.897 |
| sTNFR1 | 5250.49 ± 3930.86 | 4218.0 ± 2669.7 | 7094.2 ± 5128.1 | **0.019** |
| sTNFR2 | 700.93 ± 530.89 | 504.3 ± 314.7 | 1052.1 ± 658.7 | **0.005** |
| TWEAK | 5356.56 ± 8842.29 | 4751.4 ± 8849.7 | 6437.2 ± 9055.2 | 0.263 |
| APRIL | 115117.59 ± 90655.80 | 103390.1 ± 75841.8 | 136059.5 ± 112549.6 | 0.478 |
| BAFF | 17953.14 ± 15213.18 | 15178.8 ± 9361.8 | 22907.3 ± 21759.7 | 0.460 |
| LIGHT | 93.91 ± 239.99 | 56.5 ± 102.9 | 155.4 ± 367.5 | 0.328 |
| sCD30 | 2643.43 ± 2433.90 | 2330.0 ± 2228.0 | 3203.1 ± 2761.4 | 0.303 |
| IFNg | 23.60 ± 24.83 | 23.4 ± 20.7 | 23.9 ± 31.8 | 0.613 |
| IFNalfa2 | 40.49 ± 82.22 | 37.4 ± 84.0 | 45.6 ± 82.4 | 0.807 |
| IFNbeta | 26.60 ± 25.22 | 19.8 ± 15.7 | 36.3 ± 33.0 | 0.227 |
| IL28a | 79.44 ± 274.64 | 29.2 ± 51.3 | 168.3 ± 449.2 | 0.100 |
| IL29 | 65.55 ± 76.27 | 51.0 ± 47.9 | 89.8 ± 106.8 | 0.408 |
| sIL6R-beta | 118081.52 ± 81401.00 | 102254.7 ± 70577.8 | 146343.8 ± 93971.5 | 0.125 |
| IL1beta | 2.95 ± 3.86 | 2.5 ± 3.1 | 3.8 ± 5.0 | 0.478 |
| IL4 | 27.78 ± 26.02 | 24.4 ± 18.1 | 34.3 ± 37.0 | 0.808 |
| IL6 | 29.90 ± 52.40 | 33.2 ± 61.5 | 23.6 ± 29.8 | 0.696 |
| IL8 | 67.20 ± 95.04 | 57.2 ± 87.7 | 85.1 ± 108.0 | 0.573 |
| IL10 | 16.41 ± 15.90 | 15.1 ± 12.4 | 18.7 ± 21.2 | 0.965 |
| IL16 | 76.23 ± 125.31 | 62.8 ± 82.6 | 100.2 ± 179.9 | 0.377 |
| sILR6a | 5499.32 ± 3375.99 | 4752.8 ± 2855.8 | 6832.3 ± 3908.1 | **0.038** |
| IL11 | 3.11 ± 3.41 | 2.6 ± 2.9 | 4.0 ± 4.2 | 0.403 |
| IL12 (p40) | 33.55 ± 40.19 | 28.1 ± 25.7 | 43.9 ± 59.4 | 0.982 |
| IL12 (p70) | 15.07 ± 26.74 | 12.2 ± 22.3 | 19.8 ± 33.2 | 0.576 |
| IL19 | 231.79 ± 266.31 | 201.5 ± 192.9 | 278.3 ± 355.5 | 0.813 |
| IL20 | 39.56 ± 64.89 | 33.0 ± 45.7 | 52.2 ± 92.4 | 0.976 |
| IL22 | 61.84 ± 79.01 | 43.7 ± 32.0 | 95.3 ± 122.0 | 0.150 |
| IL26 | 2250.08 ± 2625.67 | 1878.7 ± 1598.7 | 2907.1 ± 3832.8 | 0.721 |
| IL27 | 187.22 ± 234.59 | 140.7 ± 110.7 | 257.1 ± 344.5 | 0.892 |
| IL32 | 104.68 ± 127.53 | 88.6 ± 135.5 | 132.2 ± 111.8 | 0.054 |
| IL34 | 796.09 ± 768.15 | 626.4 ± 464.1 | 1096.4 ± 1083.1 | 0.344 |
| IL35 | 209.09 ± 186.10 | 165.8 ± 140.6 | 274.1 ± 229.4 | 0.222 |
| MMP1 | 548.47 ± 1026.25 | 493.0 ± 788.2 | 619.0 ± 1307.7 | 0.979 |
| MMP2 | 1118.42 ± 1362.71 | 846.4 ± 784.1 | 1481.1 ± 1862.4 | 0.767 |
| Osteocalcin | 883.54 ± 620.69 | 804.0 ± 565.8 | 1025.5 ± 708.0 | 0.534 |
| Osteopontin | 113372.41 ± 82555.24 | 105009.3 ± 75433.5 | 128306.5 ± 95090.2 | 0.675 |
| Pentraxin | 351.42 ± 267.70 | 312.2 ± 175.2 | 421.4 ± 380.3 | 0.919 |
| TSLP | 29.27 ± 28.57 | 23.2 ± 18.0 | 40.1 ± 39.9 | 0.060 |
| sCD163 | 59394.53 ± 34319.58 | 50027.4 ± 27947.8 | 76121.6 ± 39126.1 | **0.014** |
| Chitinase3like1 | 58668.44 ± 45223.32 | 47593.7 ± 31833.8 | 78444.8 ± 58738.3 | 0.157 |

Values are expressed as ng/ml/mgProt; mean ± SD are reported. A p value < 0.05 was considered significant.

Abbreviations: CSF = cerebrospinal fluid; EDA = evidence of disease activity; NEDA = no evidence of disease activity; CCL21 = chemokine C-C motif ligand 21; CXCL13 = chemokine C-X-C motif ligand 13; CXCL5 = chemokine C-X-C motif ligand 5; CCL11 = chemokine C-C motif ligand 11; CCL24 = chemokine C-C motif ligand 24, CCL26 = chemokine C-C motif ligand 26; CX3CL1 = chemokine C-X3 -C motif ligand 1; GMCSF = Granulocyte macrophage colony-stimulating factor; CXCL1 = chemokine C-X-C motif ligand 1; CXCL2 = chemokine C-X-C motif ligand 2; CCL1 = chemokine C-C motif ligand 1; CXCL10 = chemokine C-X-C motif ligand 10; CXCL11 = chemokine C-X-C motif ligand 11; CCL2 = chemokine C-C motif ligand 2; CCL8 = chemokine CC motif ligand 8; CCL7 = chemokine C-C motif ligand 7; CCL13 = chemokine C-C motif ligand 13; CCL22 = chemokine C-C motif ligand 22; MIF = macrophage migration inhibitory; CXCL9 = chemokine C-X-C motif ligand 9; CCL3 = chemokine C-C motif ligand 3; CCL15 = chemokine CC motif ligand 15; CCL19 = chemokine C-C motif ligand 19; CCL23 = chemokine C-C motif ligand 23; CXCL16 = chemokine C-X-C motif ligand 16; CXCL12 = chemokine C-X-C motif ligand 12 or stromal cell-derived factor; CCL25 = chemokine C-C motif ligand 25; TNF = tumor necrosis factor; sTNFR1 = soluble receptor 1 of tumor necrosis factor; sTNFR2 = soluble receptor 2 of tumor necrosis factor; TWEAK = TNF-like weak inducer of apoptosis; APRIL = A proliferation-inducing ligand, or tumor necrosis factor ligand superfamily member 13; BAFF = B cell-activating factor of the tumor necrosis factor family ; LIGHT = tumor necrosis factor ligand superfamily member 14 or tumour necrosis factor ; sCD30 = soluble form of CD30; IFNg = interferon gamma; IFNalfa2 = interferon alfa 2; IL28a = interleujin-28a; sIL6R-beta = soluble receptor beta of interleukin-6; IL1beta = interleukin-1 beta; IL4 = interleukin-4; IL6 = interleukin-6; IL8 = interleukin-8; IL10 = interleukin-10; IL16 = interleukin-16; sILRa = soluble interleukine receptor a; IL12 (p70) = interleukin-12 (p70); IL20 = interleukin-20; IL22 = interleukin-22; IL 26 = interleukin-26; IL32 = interleukin-32; IL34 = interleukin-34; IL35 = interleukin-35; sCD163 = Soluble form of CD163

**Supplementary Table 2.** Logistic regression model evaluating the association of time from last relapse, BMI, administration of previous disease-modifying treatment, Chitinase3like1 and disease activity after two years of cladribine treatment.

|  | **OR** | **95%CI lower bound** | **95%CI upper bound** | **p value** |
| --- | --- | --- | --- | --- |
| **Time from last relapse** | 0.19 | 0.018 | 1.982 | 0.165 |
| **BMI** | 0.983 | 0.641 | 1.507 | 0.938 |
| **Previous DMT** | 0.158 | 0.019 | 1.29 | 0.085 |
| **Chitinase3like1** |  |  |  |  |
| Chitinase 2nd percentile | 0.047 | 0.002 | 1.114 | 0.058 |
| Chitinase 3rd percentile | 0.063 | 0.004 | 1 | 0.05 |
| Chitinase 4th percentile | 0.044 | 0.003 | 0.716 | 0.028 |

BMI = body mass index; CI = confidence interval; DMT = disease modifying therapy; OR = odds ratio.
